# A cross-country efficiency and productivity evaluation of commercial banks in South Asia: A meta-frontier and Malmquist productivity index approach

**DOI:** 10.1371/journal.pone.0265349

**Published:** 2022-04-06

**Authors:** Wasi Ul Hassan Shah, Gang Hao, Nan Zhu, Rizwana Yasmeen, Ihtsham Ul Haq Padda, Muhammad Abdul Kamal

**Affiliations:** 1 School of Management, Zhejiang Shuren University, Hangzhou, China; 2 Department of Management Sciences, City University of Hong Kong, Hong Kong, Hong Kong Special Administrative Region, China; 3 The Western Business School, Southwestern University of Finance and Economics, Chengdu, China; 4 School of Economics and Management, Panzhihua University Panzhihua, Sichuan, China; 5 Department of Economics, Federal Urdu University of Arts, Science and Technology, Islamabad, Pakistan; 6 Department of Economics, Abdul Wali Khan University Mardan, Mardan, Khyber Pakhtunkhwa Province, Pakistan; International Centre for Integrated Mountain Development (ICIMOD), Kathmandu, Nepal, NEPAL

## Abstract

South Asia primarily consists of developing economies with diverse financial systems. The commercial banking industry plays a crucial role in each country’s financial development in the region. This research aims to evaluate commercial banking industries’ efficiency and productivity growth in the South Asian (SA) region over 6 years (2013–2018). In addition, the technology gap among the banking industries of all countries is also explored. Data envelopment analysis (DEA) Meta-frontier is employed to measure the technical efficiency (TE) and technology gap ratio (TGR) among the countries. Further Malmquist productivity index (MPI) is used for productivity change estimation. Results indicate that, on average, 147 commercial banks (CBs) have a technical efficiency score of 0.6208, while CBs in Nepal are the most efficient in the region with an average score of 0.7153. The Meta frontier analysis also confirms the presence of different production technologies in CBs. Nepal’s CBs group frontier is closer to meta-frontier (technology gap ratio, TGR = 0.9361) While, Bangladesh, Pakistan, India, and Sri Lanka rank second, third, fourth, and fifth, respectively. The results of productivity contend that the total factor productivity change of all 147 CBs decreases by 0.8 percent on average over the study period. CBs have enhanced their productivity growth in Sri Lanka, Nepal, and Pakistan, but declining trends have been witnessed in Indian and Bangladesh’s commercial banking industries.

## 1. Introduction

The financial sector is one of the most important aspects of a country’s economic system since sustainable economic development is mainly dependent on its financial sector development [1]. CBs play a critical role in achieving economic development goals through the necessary fund supply [2]. A.L. Sobiech et al. [3], recognized that commercial banks are essential sources of long-term investment finance. CBs are also the backbone of the banking system since they lend capital to the private sector [4]. Indeed, these banks contribute widely to financial capital mobilization in any economy. However, the efficient growth of these banks is determined primarily by the aggressive marketing strategy in the banking sector and technological advances in the production of new products. Such factors increase the productivity and efficiency of the CBs. Consequently, the CBs’ performance assessment is essential in proposing policy outcomes to decision-makers. The fundamental intention is to assess the efficiency and productivity change of CBs in the markets where they operate and further compare their performance with their competitors. This paper analyzes the performance of South Asian CBs due to its virtual importance in the developing world. Indeed, the South Asian region has the potential to influence the world economy as it is a densely populated region with approximately 1.8 billion population. Commercial banking industries of South Asian countries are the driving force for the financial structure of these developing economies [5]. Though these countries have similar cultures, geography, and economic conditions to some extent, commercial banks’ production technologies still vary from country to country. Moreover, owing to heterogeneous technology, the performance of each country’s commercial banks may differ.

India’s commercial banking industry, for example, is one of the world’s leading and well-developed industries. The Indian banking sector continued to develop and was found to be efficient at 73.44 percent [6]. Nevertheless, CBs production technologies in Pakistan, Sri Lanka, Bangladesh, and Nepal differ from the massive Indian CB industry. However, these countries are reviving policies to strengthen the operations of the banking system, thereby banking efficiency and assets of the banks are expected to rise. Tables 1 & 2 show the financial indicators, average equity returns (AROE), and average asset returns (AROA) of South Asian banking industries, which explain the financial position of banking sectors. The Mean ROAE and ROAA of commercial banks for Nepal are the highest of sample countries.

**Table 1:** Average of returns on equity (AROE) of 147 SA CBs.

ROAE	2013	2014	2015	2016	2017	2018	2013–2018
**Mean BD**	9.4718	11.5791	11.75	11.4971	11.8035	10.515	11.1028
**Mean IN**	8.2339	8.8361	2.2114	4.5303	-4.8792	-2.9186	2.669
**Mean LK**	9.338	15.344	15.1007	16.394	16.0227	12.198	14.0662
**Mean NP**	68.6666	22.4829	29.4369	23.4886	21.3897	15.474	30.1565
**Mean PK**	7.9116	12.5489	14.3305	14.0705	10.2168	12.9495	12.0046
**Average All**	23.7208	14.1533	14.4515	13.6064	10.162	8.8282	14.1537

**Note**: BD stands for Bangladesh, IN for India, LK for Sri Lanka, NP for Nepal, and PK for Pakistan, respectively.

**Table 2:** Average of returns on assets (AROA) of 147 SA CBs.

ROAA	2013	2014	2015	2016	2017	2018	2013–2018
**Mean BD**	1.0368	1.125	1.3074	1.1903	1.0659	0.9038	1.1049
**Mean IN**	0.8131	0.8575	0.4692	0.5706	0.025	0.0725	0.468
**Mean LK**	1.0267	1.5087	1.3887	1.4713	1.5173	1.2473	1.36
**Mean NP**	1.8131	1.7457	1.984	2.0094	2.2563	1.8457	1.9424
**Mean PK**	0.9989	1.2679	1.2926	1.2021	0.8153	0.9947	1.0953
**Average All**	1.167	1.2739	1.2681	1.2687	1.1114	0.9765	1.1776

**Note**: BD stands for Bangladesh, IN for India, LK for Sri Lanka, NP for Nepal, and PK for Pakistan, respectively.

**Fig 1:**
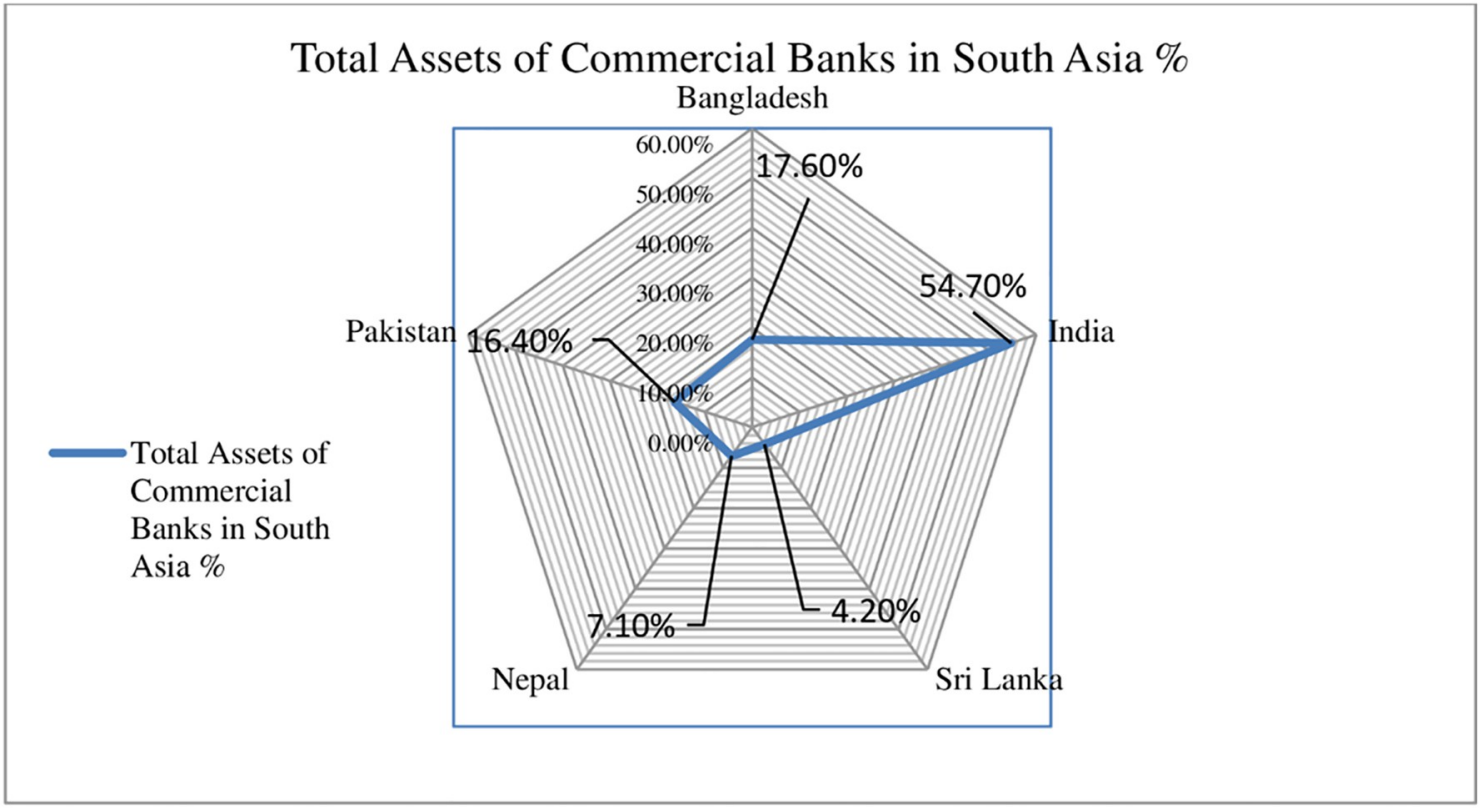
Percentage of total assets of South Asian commercial banks (Bank focus).

Fig 1 indicates the variability in South Asian banks’ total assets. The total assets of Indian commercial banks are 54.70%, followed by Bangladesh (17.60%), Pakistan (16.40%), Nepal (7.10%), and Sri Lanka (4.20%), respectively. In the extant literature, a variety of approaches such as financial ratio analysis [7], data envelopment analysis (DEA) [8], stochastic semi-nonparametric envelopment of data [9], and stochastic frontier analysis (SFA) [10], have been employed to assess the banking efficiency and productivity. However, data envelopment analysis is one of the most popular non-parametric linear programming techniques, extensively used to measure the efficiency and productivity change of banking industries [11–15]. Due to the production technology gap, limited research has been carried out on cross-country performance evaluation of commercial banking industries globally [16, 17]. For instance, many studies [18–24] have been conducted to measure the efficiency and productivity of South Asian commercial banks for individual countries. While the research conducted by [25] concentrated on pre- and post-regulatory reform efficiency of Indian and Pakistani CBs, none of the studies assessed regional performance on a combined basis and measured the technology gap ratio (TGR). To fill this void, we evaluate the efficiency and technology gap ratio of South Asian commercial banks through meta-frontier analysis, which is a significant approach to measure technological gaps between different production units or industries, [26, 27]. To best of our knowledge, this is the first study on the subject. This research further contributes to the existing literature in the following ways. Using the data of 147 CBs from Bangladesh, India, Nepal, Pakistan, and Sri Lanka, efficiency and productivity change analysis were conducted through data envelopment analysis (DEA), first, it evaluates the annual operational efficiency of all DMUs for a specific period and shows the annual trend of average technical efficiency. Secondly, using Meta-frontier we measured the technology gap ratio (TGR) in CBs of sample countries, which has never been performed before for South Asia region. Finally Malmquist productivity index is employed to measure the productivity change in sampled CBs and differentiate the main factor (efficiency change or technological change) behind the total factor productivity change.

The rest of the paper is designed as follows: section 2 includes all the relevant literature, while Section 3 methodology and data description. Section 4 presents the results evaluated through empirical analysis, while section 5 concludes the study and offers policy implications.

## 2. Literature review

Data envelopment analysis is widely used to assess banking performance using linear programming techniques despite various approaches to resolving banking efficiency problems. Several studies have been conducted to evaluate the efficiency and productivity change of banking industries around the world. Such as, [28] is the first who use DEA on different bank branches in the US. They found that six out of 14 bank branches are relatively inefficient. Halkos (2004) [11] measured the performance of the Greek banking sector by applying data envelopment analysis. They argued that the size of the total assets accompanies the efficiency of Greek banks. In contrast, the traditional DEA method [12] employed a slack-based measure network DEA model to assess the Japanese regional bank’s management efficiency. They mentioned that the medium-sized banks are incompetent. Therefore, it is a dire need to improve the management efficiency of these banks, particularly in the marketable securities division than in the lending division. Fukuyama (2013) [13] analyzed productivity change for 269 Japanese Shinkin banks by analyzing data envelopment and reported that in the case of a weak economy, the bank purposely abandoned some current lending opportunities to minimize non-performing loans reallocating capital when the economy could be expected to recover. Fukuyama (2015), [14] also conducted a study for Japanese commercial banks and developed a dynamic two-stage network production model. They examined that bank inefficiency affects the portfolio size of the assets. Recently Q.Phung et al. [29] employed the DEA on USA banking and found a negative relationship between non-performing loans and bank efficiency. Emrouznejad et al. [30] surveyed the DEA application in various industries and argued that a large part of the DEA literature had been published in connection with banking industries in different countries and regions.

Additionally, several studies are conducted to explore the same nexus for example [15] for East Virginia; [31] for Turkey; [32] for Canada; [33] for Korea; [34] and [35] for the US; [36] for the UK retail Banks; [37–39] for India; [40] for Vietnam [41–43] for China; and [44] for a panel of 17 Central and Eastern European countries. Onwards wide range of scholars applied the DEA for performance evaluation of Commercial banks. For example [45–54] applied basic CCR and BCC models of DEA in different countries around the globe to measure the operational efficiencies of CBs.

Some scholars considered undesirable outputs (non-performing loans) to evaluate the banking performance, for instance [33]; [43] and [55, 56]. Simultaneously, [34] and [57–62], followed the super-efficiency model in data envelopment analysis. However, heterogeneous factors are ignored while evaluating the baking performance. Indeed, each bank has its own production sets that might be different owing to physical and capital stocks, in conjunction with the economic and social structure [63]. The ignorance of heterogeneous factors leads to biased banking efficiency measures. Meta frontier analysis is a more suitable approach to account for the heterogeneous factors, which follows two steps. Firstly, banks are categorized according to their inner individualities (public CBs, joint-stock CBs, and foreign banks) to evaluate a group-specific production frontier for each cluster (pones). Secondly, the Meta frontier is measured by enveloping the group- particular frontiers [64, 65]. By employing the Meta-frontier analysis on Chinese commercial banking data, C.Lee et al. [66] found that fintech innovation improves the bank efficiency and enhances the technology used by particular commercial banks. Authors further decomposed the source of Meta frontier inefficiency for various banks with undesirable outputs and suggested that foreign banks do not operate efficiently in developed countries due to technology gaps of commercial banks. Considering the facts mentioned above, we also used the Meta frontier approach to account for the heterogeneous factors in South Asian CBs. Moreover, we fail to find a comprehensive study on the South Asia region and its technological gaps. Therefore, this study would be a valuable addition to the existing literature in different directions.

## 3. Research methodology

**Fig 2:**
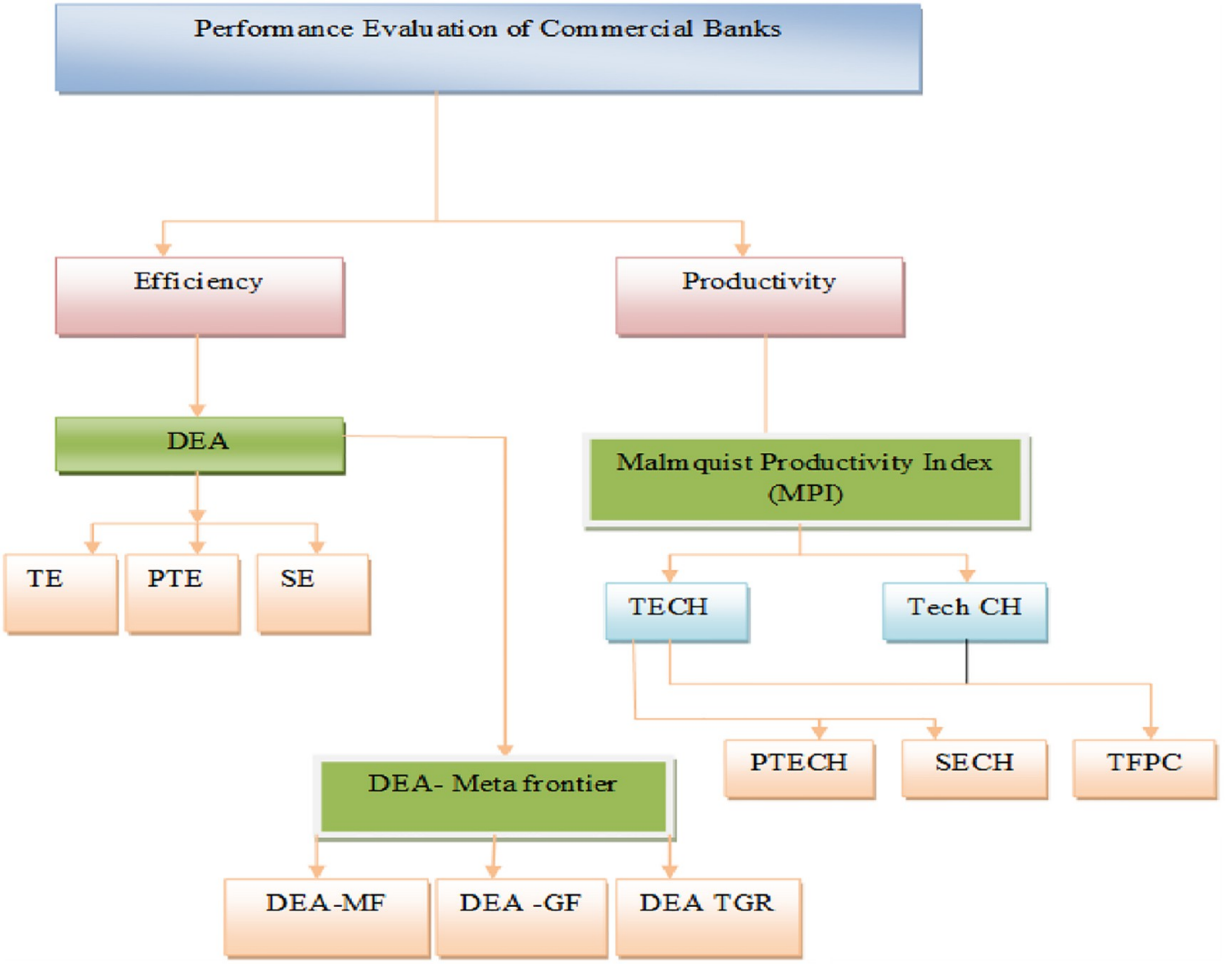
Empirical evaluation outline of South Asian commercial banks’ performance analysis.

Parametric stochastic frontier analysis (SFA) and non-parametric data envelopment analysis are two renowned techniques to gauge the efficiency of homogeneous decision-making units (DMUs). Due to relaxed normality assumption, DEA is a more powerful tool in efficiency estimation [67]. We outline three steps procedures for empirical evaluation of South Asian commercial banks’ performance analysis (see Fig 2). First, data envelopment analysis (CCR, BCC) estimates the technical efficiency of CBs. Secondly, DEA-Meta frontier is applied to find the Meta frontier, group frontier, and technological gap ratio. Finally, we used the Malmquist Productivity Index for productivity analysis.

### 3.1. CCR model

Considering a set of *J* DMUs with *n* input and *m* output in *T* (*t* = 1,…, *T*) periods. Suppose in time period *t*, decision-makers are using inputs $x^{t}\in R_{+}^{n}$ to produce outputs $y^{t}\in R_{+}^{m}$. Define the input requirement set in period *t*, which is:

$$L^{t}(y^{t})=\{x^{t}:x^{t}\;\text{can}\;\text{produce}\;y^{t}\}.$$

Assume that *L*^*t*^(*y*^*t*^) is non-empty, closed, convex, bounded and satisfies substantial disposability property of inputs and outputs *L*^*t*^(*y*^*t*^) is bounded from below by the input isoquant (a constant returns to scale (CRS) production boundary), that is:

$$\text{Isoq}L_{}^{t}\left(y^{t}\right)=\left\{x^{t}:x^{t}\in L^{t}\left(y^{t}\right),\lambda x^{t}\notin L^{t}\left(y^{t}\right)\;\text{for}\;\lambda <1\right\}.$$

Define the input distance function of period *t* as follows:

$$D_{}^{t}\left(y^{t},x^{t}\right)=\underset{\theta }{\text{sup}}\left\{\theta :\left(x^{t}/\theta \right)\in L^{t}\left(y^{t}\right),\theta >0\right\}.$$

$${\text{TE}}^{t}\left(y^{t},x^{t}\right)=1/D_{}^{t}\left(y^{t},x^{t}\right).$$ (1)

Thus, the following is the DEA-CCR model for measuring TE in time t:

TE = 1 means that a specific DMU is being evaluated compared to other DMUs, indicating that it is productively inefficient since it uses excessive inputs. In contrast, TE = 1 indicates that the DMU is entirely efficient.

### 3.2. BCC model

In addition, Banker et al. [68] devised the DEA-BCC model, which provided that TE could be further decomposed into pure technical efficiency (PTE) and scale efficiency (SE). By generating the production possibility set from observed values set of input-output bundles without taking a functional form of the production technology:

TE=PTE×SE. (2)

The BCC model represents the PTE without including the SE and considers variable returns to scale (VRS). For the PTE, the BCC model is as follows:

$${Max\;h}_{k}=\sum_{r=1}^{s}u_{r}Y_{rk}+\omega$$

Subject to:

$$\sum_{i=1}^{m}v_{i}X_{ik}=1$$

$$\overset{s}{\underset{r=1}{\sum }}\mu_{r}Y_{rj}\;-\overset{m}{\underset{i=1}{\sum }}v_{i}X_{ij}\;+\;\omega \;\leq 0$$ (3)

$$u_{r}\geq 0{;\;v}_{i}\geq 0;$$

r=1,⋯,s;i=1,⋯,m;j=1,⋯,n;

ω=free.

The DEA model becomes with increasing returns to scale when *w >* o, it becomes decreasing returns to scale when *w <* o, whereas DMU jb is Pareto-efficient in one condition, if and only if *q*b = 1, where *q*b represents pure technical efficiency. When PTE or SE is below one, it implies that a DMU is inefficient compared to other DMUs being observed. We may infer a lack of skill in converting inputs to optimal output levels. Emrouznejad et al. [30] carried out a survey and reviewed existing research from 1978 to 2016, emphasizing the development of DEA models and their real-world applications. The DEA application is currently widespread in the banking and financial sector for performance evaluation.

### 3.3. Meta frontier analysis

Meta frontier analysis can measure the technology gap between different groups of DMUs. Consistent with [63], an output-oriented measurement of technical efficiency with relation to meta-technology for one observation (or one form) can be described as:

$${TE}^{\to }\left(x,y\right)=D^{\to }(x,y)$$

For group A, the output-oriented measurement of technical efficiency about the technology of a group is described as:

$${TE}^{A}\left(x,y\right)=D^{A}(x,y)$$

Hence, the output-oriented meta-technology ratio for group A can be written as:

$${MRT}^{A}(x,y)=\frac{D^{\to }(x,y)}{D^{A}(x,y)}=\frac{{TE}^{\to }(x,y)}{{TE}^{A}(x,y)}$$

Or

$$TE^{\to }\left(x,y\right)=TE^{A}\left(x,y\right)*MRT^{A}\left(x,y\right)$$ (4)

Where *MRT*^*A*^(*x*, *y*) computes the variation between meta-frontier and the groups A frontiers, also known as the TGR.

**Fig 3:**
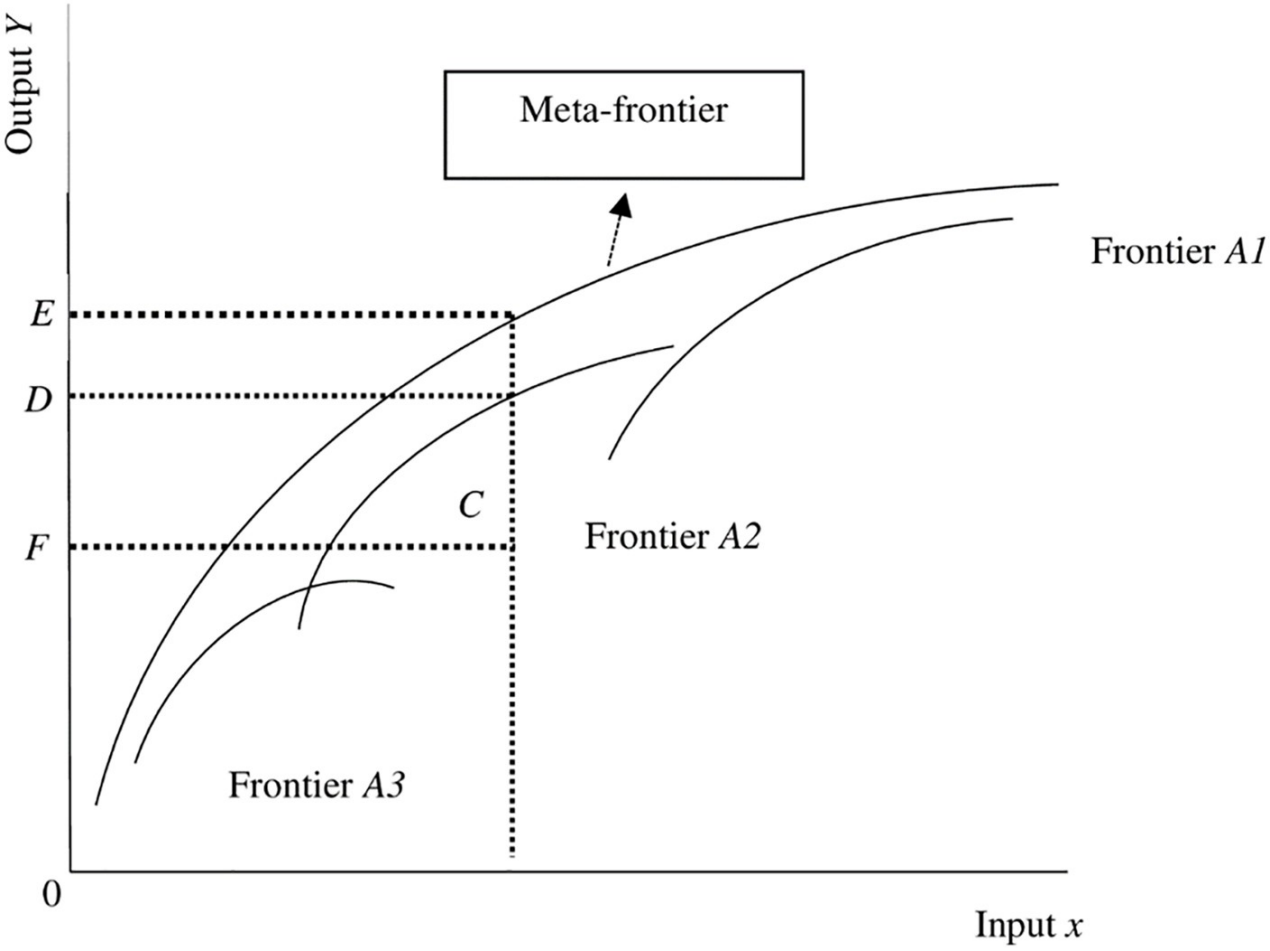
Efficiency analysis based on meta-frontier analysis.

Fig 3 depicts the meta-frontier function model and meta-technology ratio, where the meta-frontier envelops the groups A 1, 2, 3 frontiers (or country frontiers), and the meta-technology ratio is calculated by dividing $\frac{\frac{OF}{OE}}{\frac{OF}{OD}}$ which equals $\frac{{TE}^{\to }(x,y)}{{TE}^{A}(x,y)}$.

### 3.4. Malmquist productivity index

Fisher index, Malmquist productivity index (MPI, also known as Total Factor Productivity, or TFP), and Tomqvist index are widely used indices to calculate productivity change. We used MPI in this study because of its extensive use in banking literature for productivity analysis. The extensive use of MPI in banking is because it does not require cost minimization and profit maximization assumptions. In Färe & Grosskopf (1994) [69], an approach for the value of MPI is obtained as follows:

$$M({y^{t+1},x}^{t+1};y^{t},x^{t})={\left[\frac{D^{t}(y^{t+1},x^{t+1})}{D^{t}{(y^{t},x}^{t})}\times \frac{D^{t+1}(y^{t+1},x^{t+1})}{D^{t+1}(y^{t},x^{t})}\right]}^{1/2}.$$

To disintegrate productivity change into TE change and technical change, the MPI approach can be used:

$$M\left(y^{t+1},x^{t+1};y^{t},x^{t}\right)=\frac{D^{t}\left(y^{t+1},x^{t+1}\right)}{D^{t}\left(y^{t},x^{t}\right)}\times {\left[\frac{D^{t}\left(y^{t+1},x^{t+1}\right)}{D^{t+1}\left(y^{t+1},x^{t+1}\right)}\times \frac{D^{t}\left(y^{t},x^{t}\right)}{D^{t+1}\left(y^{t},x^{t}\right)}\right]}^{1/2}$$ (5)

Where $TE\;Change=\frac{D^{t}(y^{t+1},x^{t+1})}{D^{t}(y^{t},x^{t})}$, $Technical\;Change={\left[\frac{D^{t}(y^{t+1},x^{t+1})}{D^{t+1}(y^{t+1},x^{t+1})}\times \frac{D^{t}(y^{t},x^{t})}{D^{t+1}(y^{t},x^{t})}\right]}^{1/2}$.

Some ratios beyond the brackets in Eq (5) indicate the measure of TE shift between time t and time t+1. While the ratios within the brackets reflect a shift in technology, as our studied industry is commercial banking, the above equation can further elaborate on how efficiency and technology change over time. MPI can be achieved by solving a series of linear programming equations; see [69], for more details. If MPI>1 (MPI<1), the Malmquist index progress (regress) between t and t+1 is inferred. There is no noticeable difference in efficiency from time t to time t+1 if the MPI value is 1. If TEC>1, TEC<1, or TEC = 1, the technical efficiency will increase, decrease, or remain constant between periods t and t+1, respectively. TC > 1 and TC < 1 reflect progress and regress in production technology between period t and t+1, respectively. The TEC can be further decomposed into the scale efficiency change (SEC) and pure technical efficiency change (PTEC): TEC = SEC×PTEC [70, 71]. The MPI technique is frequently used in banking studies [30]. Since its inception by [72], the MPI technique has been widely applied in banking research. For example, [73–78] conducted studies on commercial bank productivity change in various parts of the world.

### 3.5. Data and descriptive- statistics

The choice of inputs and outputs variables in DEA is a major concern for researchers. Literature advocates that two different approaches are used to measure commercial bank efficiency: production and intermediate. Banks are considered service providers in the production approach, where they emphasize operating costs and count deposits as output without taking into account interest expenses paid on deposit collection. On the contrary, the intermediation approach used deposits as an input variable to produce more bank assets, while all operating costs and interest expenses were used as input. Production approaches are more applicable for branch-level data, while intermediation approaches are for bank-level data. Data for two inputs (Interest expenses, Non-interest Expenses) and two outputs (Net interest income, Non-interest income) were collected from the Bank focus website. The data includes 147 commercial banks from five South Asian countries (Bangladesh, India, Sri Lanka, Nepal, and Pakistan) for the time 2013–2018. The panel includes 35 CBs from Bangladesh, 40 from India, 16 from Sri Lanka, 36 from Nepal, and 20 from Pakistan. DEA-Max software was used for efficiency and productivity analysis. Table 3 presents the descriptive statistics for the variables.

**Table 3:** Descriptive statistics of input-output variables (N = 147).

Variables	Interest expenses	Non-interest expenses	Net interest income	Non-interest income
Max	7333797	3625484	6974456	2396662
Min	240	157	245	80
Average	596569	251382	369409	155797
SD	1188127	493071	819425	341387

**Note**: SD shows standard deviation; Max and Min designate maximum and minimum values, respectively. All input-output variables are presented in real values of thousand US dollars.

## 4. Results and discussions

DEA method is applied to measure the operating efficiency of South Asian region banks. All CBs ’operational efficiency scores are reported in Table 4. The results suggest that the average TE of all 147 CBs is 0.6208, which indicates that there is still 0.3792 percent technical inefficiency in these CBs’ operations. In other words, South Asian banks can improve their technical efficiency by reducing the input amount by 37.92 percent to generate the same output. Further mean PTE and SE scores are 0.7022 and 0.8917 for all CBs. TE and PTE scores of all CBs were higher in 2015, while SE scores were higher in 2013.

**Table 4:** Years-wise mean operational efficiency scores of all 147 CBs.

Years	TE	PTE	SE
2013	0.6323	0.6864	0.9300
2014	0.5271	0.6492	0.8336
2015	0.6702	0.7282	0.9250
2016	0.6654	0.7392	0.9034
2017	0.6223	0.7036	0.8901
2018	0.6077	0.7068	0.8679
Mean 2013–2018	0.6208	0.7022	0.8917

**Note**: TE shows technical efficiency, PTE Shows pure technical efficiency, and SE shows scale efficiency.

**Fig 4:**
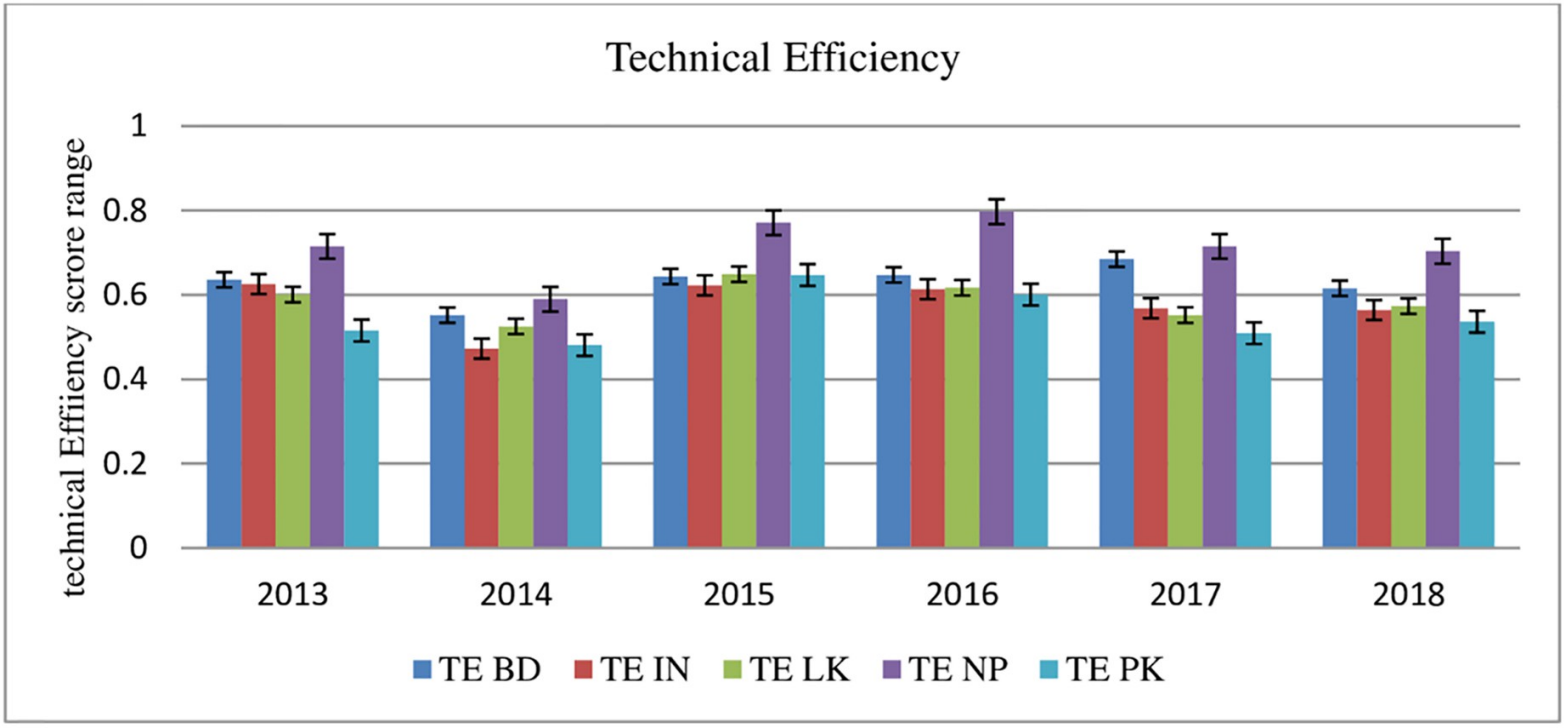
Technical Efficiency of CBs across the South Asian countries over time.

Table 5 ranks the CBs of all the countries on the performance based on TE. Mean OE scores (TE, PTE, and SE) indicate that 36 Nepalese CBs perform better than their counterparts in the 2013–18 period. These efficiency scores of Nepalese CBs are aligned with the study’s results of Gajurel et al. [79]. Fig 4 shows the average technical efficiency of CBs for each South Asian country over the study period.

**Table 5:** Mean operational efficiency scores of each country’s CBs (2013–2018).

South Asian CBs	TE	PTE	SE	Rank
Mean 35 BD	0.6299	0.6781	0.9330	2
Mean 40 IN	0.5779	0.7400	0.7925	3
Mean 16 LK	0.5863	0.6732	0.8747	4
Mean 36 NP	0.7153	0.7488	0.9586	1
Mean 20 PK	0.5484	0.6082	0.9108	5

**Note**: BD stands for Bangladesh, IN for India, LK for Sri Lanka, NP for Nepal, and PK for Pakistan, respectively.

Table 6 shows the results of DEA-MF, which indicate that average TEs of DEA-GF are higher than DEA-MF, where DEA-MF envelops all the CBs from 5 South Asian countries. Mean TEs DEA-MF declined from 61.85 percent in 2013 to 59.85 percent in 2018. Nepal’s average DEA-MF is about 71.50 percent, leading the region over a specific period. In contrast, TEs DEA-MF scores of CBs for India, Sri Lanka, Bangladesh, and Pakistan fluctuated from 54% to 62%. On the other hand, average TEs DEA-GF improved from 74.26 percent in 2013 to 76 percent in 2018. Comparing the performance of CBs operating in all 5 SA countries, we found that TE’s DEA-GF performance of CBs improved in India, Bangladesh, and Sri Lanka, while CBs performance of Nepal and Pakistan gradually decreased over the period. DEA-TGR results indicate that maximum TGR is in Nepal and then in Bangladesh CBs, approximately 94 percent and 89 percent respectively. This portrays that CBs in Nepal are operating in the best level of production technology in the South Asian region. Our results are consistent with the study conducted by [80] They measured the cost efficiency and technology gap ratio of commercial banks in 9 low-income countries for 2011–2017, and found that CBs of Nepal are cost-efficient and TGR is highest among all 9 low-income countries. In addition, Pakistan, India, and Sri Lanka are ranked 3rd, 4th, and 5th, with an average TGR of 78 percent, 77 percent, and 70 percent, respectively, indicating greater technical gaps.

**Table 6:** Technical efficiency and technology gap ratio of DEA-meta frontier in South Asian countries.

Bank Group	2013	2014	2015	2016	2017	2018	average
**Technical efficiency relative to meta-frontier (DEA-MF)**							
Mean 35 BD	0.6358	0.5519	0.6436	0.6477	0.6849	0.6157	0.6299
Mean 40 IN	0.6255	0.4727	0.6227	0.6138	0.5686	0.564	0.5779
Mean 16 LK	0.6008	0.5254	0.6492	0.6172	0.552	0.5732	0.5863
Mean 36 NP	0.7151	0.5897	0.771	0.7972	0.715	0.7034	0.7153
Mean 20 PK	0.5156	0.4809	0.6473	0.6007	0.5094	0.5362	0.5484
All Countries	0.6185	0.5241	0.6667	0.6553	0.6059	0.5985	0.6115
**Technical efficiency relative to group frontier (DEA-GF)**							
Mean 35 BD	0.6666	0.6632	0.715	0.7304	0.7811	0.7098	0.7110
Mean 40 IN	0.7437	0.7603	0.7151	0.7585	0.7532	0.7746	0.7509
Mean 16 LK	0.8031	0.8055	0.8609	0.7966	0.8497	0.8747	0.8317
Mean 36 NP	0.7491	0.7626	0.7995	0.8149	0.7304	0.7372	0.7656
Mean 20 PK	0.7507	0.5937	0.6873	0.7088	0.8180	0.7037	0.7104
All Countries	0.7426	0.717	0.7555	0.7618	0.7864	0.7600	0.7539
**Technology gap ratio (DEA-TGR)**							
Mean 35 BD	0.9562	0.843	0.8998	0.8843	0.8853	0.8644	0.8888
Mean 40 IN	0.8441	0.6211	0.8714	0.8073	0.7513	0.7282	0.7706
Mean 16 LK	0.7456	0.6492	0.751	0.7724	0.6485	0.6547	0.7036
Mean 36 NP	0.9526	0.775	0.9679	0.9792	0.983	0.9589	0.9361
Mean 20 PK	0.6838	0.8321	0.9381	0.8418	0.6265	0.7577	0.7800
All Countries	0.8364	0.744	0.8856	0.857	0.7789	0.7927	0.8158

**Note**: BD stands for Bangladesh, IN for India, LK for Sri Lanka, NP for Nepal, and PK for Pakistan, respectively.

Table 7 reveals that the total factor productivity change of all 147 CBs decreases by 0.8 percent on average. Further decomposing the total factor productivity change into efficiency change and technological change, 0.6 percent and 0.2 percent decline were noticed respectively. The results of annual growth change show that except for the years 2014/2015 and 2015/2016, there has been a decline in total factor productivity change in all other years.

**Table 7:** Malmquist Productivity Index (MPI) results over time.

years	effch	techch	pech	sech	tfpch
2013–2014	0.833	1.195	0.943	0.884	0.996
2014–2015	1.283	0.798	1.139	1.126	1.024
2015–2016	0.993	1.014	1.017	0.976	1.006
2016–2017	0.933	1.049	0.95	0.982	0.979
2017–2018	0.979	0.977	1.004	0.975	0.956
Mean 2013–18	0.994	0.998	1.008	0.986	0.992

**Note**: effch Show efficiency change, techch shows technology change, pech shows pure efficiency change, sech shows scale efficiency change, and tfpch shows total factor productivity change.

**Fig 5:**
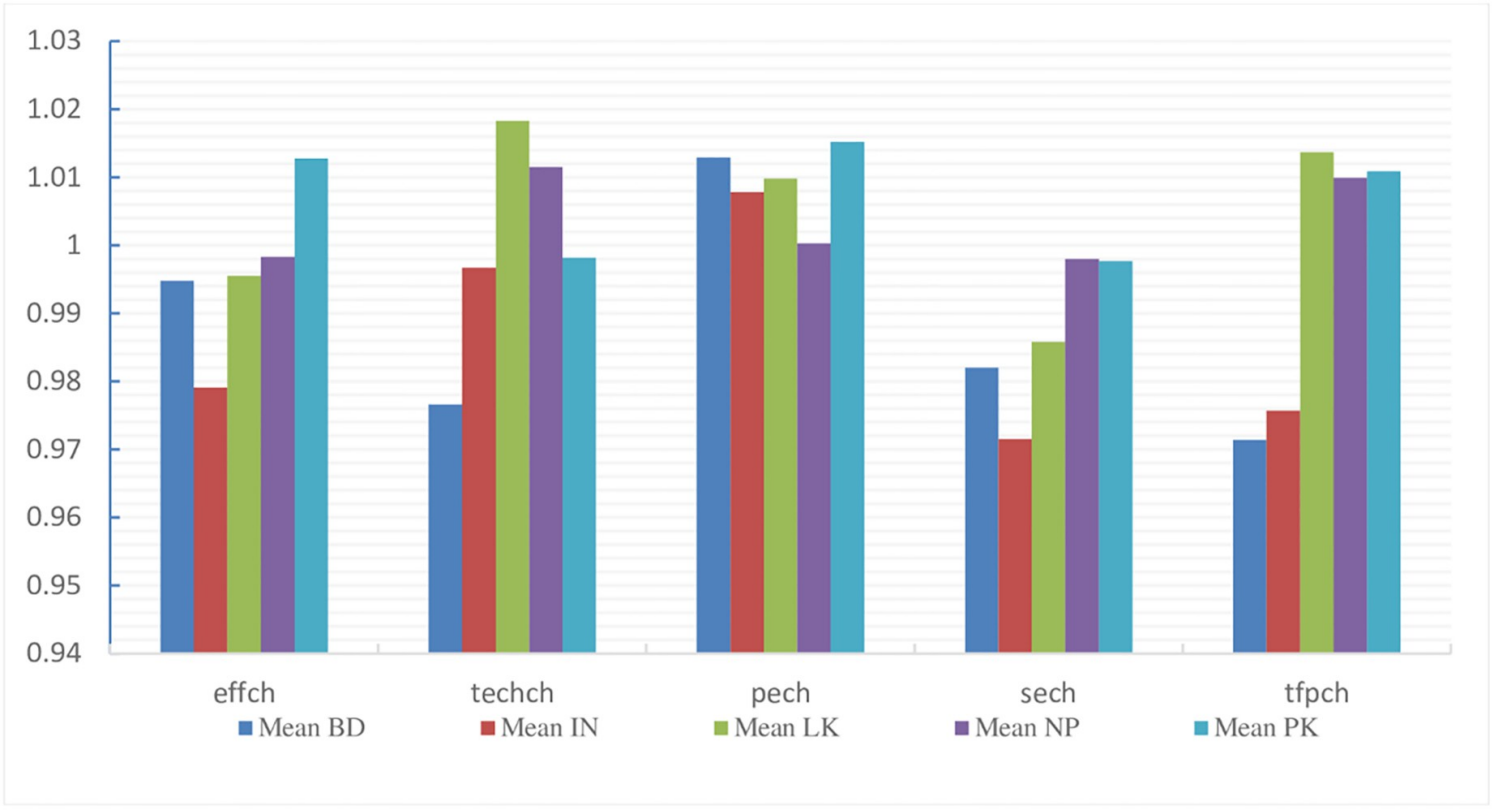
Mean productivity change in all 5 countries.

Table 8 outlines the separate productivity growth results of CBs in each South Asian country. Findings demonstrate that over the period 2013–2018, on average, CBs in Sri Lanka, Nepal, and Pakistan increased their productivity growth by 1.37%, 0.99%, and 1.09%. Still, Bangladesh and Indian CBs observed a decline in growth by 0.8% and 2.8%. Results indicate clearly that growth in total factor productivity change in CBs in Pakistan is due to increased efficiency change, which opposes the results of Zhu et al. [22] for the different study periods. In comparison, CBs growth in Sri Lanka and Nepal was mainly due to technical changes. Hence, we conclude that there is a decrease in the overall productivity growth of South Asia’s 147 CBs on average, except a slight growth observed in 2014–15 and 2015–16. Sri Lankan CBs recorded most growth in TFPC, with Pakistani and Sri Lankan ranked 2^nd^ and 3^rd^ while Bangladeshi and Indian CBs noticed a decline in their average growth. Fig 5 demonstrates the total factor productivity charge and its component technology and technical efficiency change in commercial banking industries for each South Asian country over the study period.

**Table 8:** Productivity change of each country’s CBs (2013–2018).

2013–2018	effch	techch	pech	sech	tfpch
Mean All	0.994	0.998	1.008	0.986	0.992
Mean BD	0.9948	0.9766	1.0129	0.982	0.9714
Mean IN	0.9791	0.9967	1.0078	0.9715	0.9757
Mean LK	0.9955	1.0183	1.0098	0.9858	1.0137
Mean NP	0.9983	1.0115	1.0003	0.998	1.0099
Mean PK	1.0128	0.9982	1.0152	0.9977	1.0109

**Note**: BD stands for Bangladesh, IN for India, LK for Sri Lanka, NP for Nepal, and Pk for Pakistan respectively.

## 5. Conclusion

This paper investigates the efficiency, production technology gap, and productivity growth among 147 CBs of South Asian countries from 2013–2018. Starting with financial indicators of CBs, various techniques such as DEA (CCR, BCC), DEA meta-frontier and MPI are employed for the empirical analysis. The financial indicators AROE and AROA reveal that, on average, the performance of CBs gradually decreased from 2013 to 2018. CBs in Nepal performed exceptionally well, while CBs recorded a declining trend in India. In the rest of the three sample countries (Pakistan, Sri Lanka & Bangladesh), CBs observed fluctuations in their financial performance. The DEA (CCR, BCC) results indicate that on average TE of all 147 CBs is 0.6208, depicting that there is still 0.3792 percent technical inefficiency in these CBs’ operations. In other words, South Asian banks can improve their technical efficiency by reducing input by 37.92 percent to generate the same output. Mean PTE and SE scores of all CBs are recorded as 0.7022 and 0.8917. Among the sample countries, CBs in Nepal were most efficient among with an average TE score of 0.7153. The country-wise analysis of TGR reveals that CBs of Nepal utilized the best technology (TGR, 0.9361). At the same time, Bangladesh, Pakistan, India, and Sri Lanka are ranked 2^nd^, 3^rd^, 4^th^, and 5th, respectively, as the gaps between group frontier and meta-frontier are larger.

According to the MPI results, the total productivity change showed a declining trend on average in all observed years for 147 CBs. While further decomposing the tfpch into tech and tecch, it was observed that the decline in tfpch was mainly due to the decline both in tech and tecch, however, TE change plays a significant role. CBs in Sri Lanka, Pakistan, and Nepal gained productivity growth. In Pakistan tfpch was due to tech growth, while in Sri Lanka and Nepal, CBs productivity growth was due to the technology change. CBs in the rest of two countries (Bangladesh, India) had a decline in tfpch over the observed period. Based on the obtained results this study offers valuable policy implications. Policymakers in the South Asian region must strive to develop strategies, follow CB regulations in Nepal, and seek to use the best technology available to enhance their ability to compete with other CBs in the region.

Furthermore, CBs in Bangladesh and India need to improve technical efficiency and technological change to improve productivity. In contrast, CBs in Nepal and Sri Lanka need to focus on improving their technical efficiency, similarly tfpch in Pakistani CBs was mainly due to tech, so there is always a deficiency in tecch, which could be improved to foster the change in productivity. As data were collected for 2013–2018, data availability is a limitation of our study. In the case of data availability, the impact of the 2009 financial crisis on efficiency and productivity growth could be estimated, which is an additional contribution to banking literature.
